# 精准医学时代的肺癌外科治疗走向

**DOI:** 10.3779/j.issn.1009-3419.2016.06.02

**Published:** 2016-06-20

**Authors:** 锋维 谭, 宁 李, 树庚 高, 捷 赫

**Affiliations:** 100021 北京，中国医学科学院肿瘤医院胸外科 Department of Thoracic Surgery, Cancer Institute and Hospital, Peking Union Medical College and Chinese Academy of Medical Sciences, Beijing 100021, China

**Keywords:** 精准医学, 肺肿瘤, 基因组, 蛋白组, Precision medicine, Lung neoplasms, Genome, Proteomics

## Abstract

精准医学是根据每个人从宏观到微观层面的个体差异，制定最为合适的个性化治疗方案。基因组、蛋白组、代谢组等海量生物学数据及大数据分析方法是精准医学模式的精髓。精准医学为人类攻克肿瘤带来了希望。肺癌是对人类危害最大的肿瘤。本文就肺癌的外科治疗在精准医学时代发展的方向进行了综述。

## Abstract (en)

Precision medicine is to developing the most appropriate individualized treatment for each patient based on the macro to the micro level of individual differences. Genomic, proteomics, metabolomics data, and other big data analysis methods are the essence of precision medicine. Precision medicine brings the hope to overcome cancer. Among all kinds of tumors, lung cancer is the biggest threat to human. This paper reviewed the development of precision medicine in the surgical treatment of lung cancer.

精准医学的概念于2011年11月在美国医学院发表的“迈向精准医学”（toward precision medicine）一文中首次被提出。2015年1月美国总统奥巴马在国情咨文中宣布了启动精准医学计划；2016年，中国也成立了国家精准医疗战略专家委员会，启动了中国精准医疗项目。自此，精准医学研究的帷幕全面拉开，生物医学已经逐步迈入精准医疗的时代。肿瘤是精准医学的重中之重和近期目标^[1]^。肺癌是全球死亡率第一的肿瘤，精准医学为提高肺癌的疗效带来新的契机，将使我们更加接近于攻克肺癌。

## 1 肺癌精准医学的内涵

精准医学是充分考量病人的个体化差异，针对个人或特定人群疾病开展的预防、诊断、治疗的新模式。具体到肺癌的精准医学，是指收集并整合患者的基因组、转录组、蛋白组和代谢组等遗传及分子生物学特征，结合其临床特点、生命体征、影像学表现、病理类型乃至其生活习惯和生活环境交互的所有信息，运用大数据的分析方法，找到最适合于每一个个体的治疗靶点和治疗手段，实现精准治疗，从而达到提高肺癌疗效的目标。

人类基因组计划的完成和蛋白质组、转录组、代谢组等海量分子生物学数据的产出是实现精准医学的基石，二代测序技术为代表的先进检测技术及大数据分析方法的发展是推动Proteomics精准医学发展的动力。有部分学者认为基因突变和靶向治疗就是区别于传统治疗方法的精准医学。在肺癌领域，靶向治疗取得了很多突破性的进展，尤其是针对表皮生长因子受体酪氨酸激酶（epidermal growth factor receptor-tyrosine kinase, EGFR-TK）突变^[2]^和棘皮动物微管相关蛋白4-间变性淋巴瘤激酶（echinoderm microtubule associated protein like 4-anaplastic lymphoma kinase, *EML4-ALK*）融合基因^[3]^的靶向治疗，明显提高了中晚期肺癌的疗效。但是目前已知驱动基因的非小细胞肺癌（non-small cell lung cancer, NSCLC）比例仍不足50%，并且靶向治疗对患者生存时间的提高也远未达到接近治愈肺癌的预期。因此，在深入理解肺癌分子机制、探寻更多驱动基因的同时，运用精准医学理念，在基因突变等生物标记物特征图谱的指导下，将外科治疗、化疗、放疗、靶向治疗和免疫治疗等手段个体化的实施到不同的患者，做到在合适的时间，对合适的病人实施合适的治疗，是目前肺癌精准医学最为合适的模式。

## 2 外科在肺癌精准诊断中的作用

精准诊断是实施精准医学的重要保障。传统的影像学诊断和病理诊断已经满足不了精准医疗的需求。目前，肺癌的精准诊断，应该在传统诊断方法之外，检测肿瘤特异性基因突变和特征性蛋白质、RNA、代谢产物分子标记物。这些基于生物学标本的精准检测应该体现在患者诊治的每一个关键节点，贯穿于整个病程的始终。足量并且高质量的生物学标本是完成精准诊断的保证。目前循环肿瘤细胞（circulating tumor cells, CTCs），循环游离DNA（circulating cell-free DNA, cfDNA）的检测技术发展迅猛，“液体活检”的概念也横空出世，但受其丰度低、检测技术要求高的局限，大规模应用于临床为时尚早。穿刺活检也存在假阴性比例偏高，肿瘤组织获取量不足以完成某些精准检测的局限。而随着微创技术的发展和广泛应用，手术活检给病人带来的创伤也越来越小。因此，在一定的时期内，外科手术活检对一部分患者仍然是精准诊断的有力保证。

## 3 肺癌精准治疗中外科发展方向

目前，肺癌的治疗手段包括外科、化疗、放疗、靶向治疗、免疫治疗等。化疗作为非选择性全身治疗，对部分患者疗效好，但整体缓解率仍不足50%；放疗对可手术的NSCLC疗效仍有待验证；靶向治疗只对已确定驱动基因突变的患者有效，且容易产生耐药，平均耐药时间为7个月-10个月；免疫治疗发展迅速，但距离临床常规应用还为时尚早。因此，目前外科手术切除仍然是根治肺癌的唯一可能方法，也是可切除肺癌的首先治疗手段。可以预计，在相当长的时间内，外科治疗在肺癌的治疗中将占有重要地位。

肺癌的外科治疗要体现精准医学的理念，首先要实现从艺术外科向数字外科的转变。外科手术的数字化，是精准医学的要求，也是外科自身发展的前提。在精准医学时代，每一个肺癌患者都将建立自己的信息库，涵盖从临床症状、生命体征、影像学特征等一切宏观临床信息到基因组、蛋白组、代谢组等所有微观生物学信息。将外科手术操作规范化、流程标准化、信息数据化，将术前准备、术中操作、围手术期康复、术后复查、复发监测、预后预测等外科涉及的全过程量化并且数据化，加入患者的信息库，与其他所有临床及生物学数据一起运用大数据的分析方法进行分析处理，指导外科治疗的每一个过程，在外科治疗的每一个关键节点做出对患者最合适的选择，实现外科治疗的精准化。

确定精准的切除范围是肺癌精准外科治疗的另一个方向。目前，肺叶切除仍然是是NSCLC外科治疗的标准术式。但是对于早期NSCLC，肺叶切除有可能会带来不必要的肺功能损失，目前包括楔行切除、肺段切除、解剖性部分肺叶切除等术式在内的亚肺叶切除，已经初步被证明对早期肺癌能获得与肺叶切除相似的根治性^[4, 5]^。以目前胸外科技术发展，精确的亚肺叶切除完全可以实现，所以精确挑选出适合亚肺叶切除的患者是目前亟需解决的科学问题。以肿瘤大小、影像学特征、术中冰冻病理^[6]^等作为指标来筛选亚肺叶切除的患者都有一定的局限性，对部分患者可能达不到跟肺叶切除等同的根治性。根据精准医学的理念，通过运用大数据的分析方法，将患者所有的临床信息、遗传学和生物学指标进行整合分析，使精确挑选出适合亚肺叶切除的患者、精确的确定切除范围及淋巴结清扫范围成为了可能。

外科治疗和其他治疗手段的精确协同，是实现肺癌精准治疗的重要手段。因为肿瘤本身的生物学复杂性和异质性，单凭一种治疗手段很难达到满意的疗效。肺癌也不例外，即使Ⅰ期NSCLC，术后也有高达25%的患者复发或死亡。因此，通过精准医学模式，精确的挑选出有复发风险的患者，并针对不同的患者将外科治疗、化疗、放疗、靶向治疗、免疫治疗等多种手段进行针对性的组合，是提高早期肺癌治愈率的关键所在。对于中、晚期NSCLC患者，目前以全身治疗为主。随着靶向治疗、免疫治疗等全身治疗方法的迅速发展，晚期肿瘤不行手术治疗的传统观点不断受到挑战。晚期卵巢癌的肿瘤减灭术已经常规应用于临床，转移性肾癌的原发灶减瘤术、转移性结直肠癌的原发及转移灶切除也已经被证明能延长患者生存并写入了各自的美国国家综合癌症网（National Comprehensive Cancer Network, NCCN）诊疗指南。关于晚期肺癌的减瘤术是否能让患者获益，也是目前研究的热点。对于伴随寡转移的可切除性NSCLC，外科治疗也已经被证明能给患者带来获益^[7]^。随着肺癌靶向治疗和免疫治疗的突破性进展，肺癌的减瘤术或者耐药病灶切除术也将为部分患者带来获益。运用精准医学模式，将外科治疗精准的融入到中晚期肺癌患者的全身治疗当中，是提高中晚期肺癌疗效的有效途径。

综上所述，精准医学模式的出现为提高肺癌疗效带来了无限可能。贯彻精准医学的理念，在基因突变、蛋白质、RNA、代谢产物等各种生物标记物及大数据分析方法的指导下，实施精准的外科切除、将外科治疗与其他治疗手段进行精准组合协同，是实现肺癌精准治疗的重要手段。
